# Ultrasound-assessed diaphragm dysfunction predicts clinical outcomes in hemodialysis patients

**DOI:** 10.1038/s41598-022-20450-x

**Published:** 2022-10-03

**Authors:** Jing Zheng, Qing Yin, Shi-yuan Wang, Ying-Yan Wang, Jing-jie Xiao, Tao-tao Tang, Wei-jie Ni, Li-qun Ren, Hong Liu, Xiao-liang Zhang, Bi-Cheng Liu, Bin Wang

**Affiliations:** 1 Institute of Nephrology, Zhong Da Hospital, Southeast University School of Medicine, No. 87, Dingjiaqiao Road, Gulou District, Nanjing, Jiangsu Province China; 2 Department of Gerontology, Zhong Da Hospital, Southeast University School of Medicine, Nanjing, Jiangsu China; 3 Department Epidemiology and Health Statistics, Southeast University, Nanjing, Jiangsu China; 4 Department of Ultrasound Medicine, Zhong Da Hospital, Southeast University School of Medicine, Nanjing, Jiangsu China; 5 Covenant Health Palliative Institute, Edmonton, AB Canada

**Keywords:** Nephrology, Risk factors

## Abstract

Skeletal muscle atrophy is prevalent and remarkably increases the risk of cardiovascular (CV) events and mortality in hemodialysis (HD) patients. However, whether diaphragm dysfunction predicts clinical outcomes in HD patients is unknown. This was a prospective cohort study of 103 HD patients. After assessment of diaphragm function by ultrasonography and collection of other baseline data, a 36-month follow-up was then initiated. Participants were divided into diaphragm dysfunction (DD+) group and normal diaphragm function (DD−) group, according to cutoff value of thickening ratio (i.e. the change ratio of diaphragm thickness) at force respiration. The primary endpoint was the first nonfatal CV event or all-cause mortality. A secondary endpoint was less serious CV events (LSCEs, a composite of heart failure readmission, cardiac arrhythmia or myocardial ischemia needed pharmacological intervention in hospital). 98 patients were eligible to analysis and 57 (58.16%) were men. 28 of 44 patients(63.64%) in DD+ group and 23 of 54 patients (42.59%) in DD− group had at least one nonfatal CV event or death (*p* = 0.038). Compared to DD− group, DD+ group had significantly higher incidence of LSCEs (21 *vs.*14, *p* = 0.025) and shorter survival time (22.02 ± 12.98 months *vs.* 26.74 ± 12.59 months, *p* = 0.046). Kaplan–Meier analysis revealed significantly higher risks of primary endpoint (*p* = 0.039), and LSCEs (*p* = 0.040) in DD+ group. Multivariate hazard analysis showed that DD+ group had significantly higher risk of primary endpoint [hazard ratio (HR) 1.59; 95% confident interval (CI) 1.54–1.63], and LSCEs (HR 1.47; 95%CI 1.40–1.55). Ultrasound-assessed diaphragm dysfunction predicts clinical outcomes in HD patients.

Trial registration: This study was registered with Chinese Clinical Trials Registry (www.chictr.org.cn) as ChiCTR1800016500 on Jun 05, 2018.

## Introduction

Skeleton muscle atrophy is a common body composition abnormality in patients with chronic kidney disease (CKD), especially end stage renal disease^1,2^. Low muscle mass or strength is associated with poor physical function, frailty, and higher risk of all-cause mortality in CKD patients^3–6^. Furthermore, individuals who have low muscle mass are more likely to have cardiovascular (CV) events, such as coronary artery disease, cerebrovascular disorders, heart failure, peripheral artery disease, arrhythmias^7–10^. CV diseases are the principal complication in CKD patients, and CV mortality increases as glomerular filtration rate decreases^11^. Therefore, skeleton muscle atrophy maybe an important target to improve the clinical outcomes in hemodialysis (HD) patients.

Diaphragm is the primary respiratory muscle of human body and accounts for 70% work of inspiration^12^. Diaphragm dysfunction is associated with dyspnea, decreased exercise endurance and quality of life^13^. Ultrasound is considered as an optimal tool to evaluate the diaphragmatic thickness and function in multiple diseases because of its high sensitivity and specificity, combined with zero radiation and low cost^14–17^. Ultrasound-assessed diaphragm dysfunction is reliable in early identifying diaphragm atrophy and stratifying patients’ risk of worse prognosis^18–22^. Our research group has previously reported that the ultrasound-assessed diaphragm function of HD patients is worse than that of healthy individuals, and is associated with dyspnea, fatigue and hiccup in HD patients^23^.

However, the relationship of diaphragm function and occurrence of CV events and mortality in HD patients remains unknown. The purpose of this prospective longitudinal study is to identify whether ultrasound-assessed diaphragm dysfunction predicts the clinical outcomes in HD patients.

## Methods

### Study cohort, baseline data and medical history collection

The study was conducted at the Institute of Nephrology, Southeast University, an academic medical HD center in Nanjing, Jiangsu, China. This cohort came from a previous study conducted by our team and the detail of these patients have been published^23^. A total of 103 maintenance HD patients, aged 24 to 81 years old, were recruited from May 2018 to November 2018. Follow up work started from the date of enrollment for 36 months.

According to the following assumption: 3 years follow-up, 20% probable loss during follow-up, the 3 years rate of primary endpoint in CKD with low sarcopenia score of 20%, and events ratio between high sarcopenia score group and low sarcopenia score group of 3 based on the study conducted by Hanatani et al.^7^, we calculated the minimal sample size (N = 97) with a power test (α = 0.05; power 0.9) by software PASS15(NCSS, LLC, Kaysville, Utah, USA).

Demographic features, comorbid diseases and drugs associated with CV events were recorded through detailed interview after obtaining written informed consent. CV diseases included coronary artery disease, peripheral vascular disease, stroke and arrythmia. The serum level of fasting blood-glucose (FBG), hemoglobin, albumin, low density lipoprotein cholesterol (LDLC) and left ventricular ejection fraction (LVEF, evaluated by modified Simpson method) of the recruited patients were collected, and the results within 1 month closest to enrollment were used.

### Evaluation of diaphragm function

Ultrasound was used to evaluate diaphragm function of HD patients. Measurement was taken after dialysis to minimize the impact of water accumulation. Right hemidiaphragm was used, which can be more conveniently and reproducibly measured in comparison to the left^24^. During the process of ultrasound examination, participants remained supine position^25,26^ and breathed according to ultrasound technician’s instructions. A 6–13 MHz linear high-frequency ultrasound transducer with B-mode was placed perpendicular to the chest wall on the midaxillary line at diaphragmatic zone of apposition near costophrenic angle. The diaphragm thicknesses at functional residual capacity (FRC), tidal volume (VT), residual volume (RV) and total lung capacity (TLC) were recorded, respectively. Muscle thickness of diaphragm (Tdi) was a hypoechoic structure between the bilateral hyperechoic structures (pleura and peritoneum) and final value was averaged over three continuous respiratory cycles. Diaphragm excursions (DE), the distances diaphragm motioned from cephalad side to caudal side at eupnea and forced respiratory maneuver, were measured by a 1–5 MHz M-mode ultrasound transducer. Consequently, velocity of diaphragm excursion was obtained from dividing DE by motioned time, which also averaged over three continuous respiratory cycles. Tdi and mobility of diaphragm has been associated with pulmonary function^27^. Change of Tdi (ΔTdi) at eupnea was the absolute difference between Tdi_FRC_ and Tdi_VT_ values. ΔTdi at forced respiratory was calculated by Tdi_TLC_ minus Tdi_RV_. Change ratio of Tdi at forced respiratory, termed as thickening ratio, was calculated by the following formula: (Tdi_TLC_ − Tdi_RV_)/Tdi_RV_, which reflects the maximum contractile capability of diaphragm. Thickening ratio at forced respiration has been also associated with lung function. It can help clinicians identify patients at high risk of nocturnal hypoxia, who hadn’t obvious clinical symptom related to hypoxia at early stage^28^. Therefore, thickening ratio at forced respiration might be an early indicator of diaphragm dysfunction.

### Study endpoints collection

Events collection were conducted every 2 months, either face-to-face or telephone interviews until up to 36 months. If the patients had hospitalization or received outpatient treatment in our hospital during the visit interval, we also manually checked the electronic medical records system to verify any nonfatal CV event or the causes of death. If electronic medical records were not available, we would review the discharge records or outpatient records. The primary endpoint was a composite of first nonfatal CV event and all-cause mortality. Causes of deaths were categorized as CV deaths or non-CV deaths. CV deaths included sudden death, which caused by CV events in nature unless non-CV causes were defined. Considering that some patients had more than one adverse CV event, we subsequently divided the primary endpoint into three subgroups to explore which subgroup may play a major role. These three subgroups were as follows: major adverse CV events (MACEs, a composite of death from any cause, nonfatal myocardial infarction, and nonfatal stroke), other major adverse CV events (MACEs+, a composite of hospitalization for new-onset peripheral vascular disease, coronary revascularization, and unstable angina) and less serious CV events (LSCEs, a composite of heart failure readmission, cardiac arrhythmia or myocardial ischemia needed pharmacological intervention in hospital). The secondary endpoints were MACEs; MACEs+; and LSCEs.

### Ethics

This study was approval by the ethics committee of Zhong Da Hospital affiliated to Southeast University and the study was registered with Chinese Clinical Trials Registry (www.chictr.org.cn, identification number ChiCTR1800016500, 05/06/2018). The protocol of this study was performed in accordance with the Declaration of Helsinki and relevant regulations. The informed consent was obtained from all participants.

### Statistical Analyses

All participants were divided into two groups—diaphragm dysfunction (DD +) group and normal diaphragm function (DD−) group, based on the cutoff values of thickening ratio at forced respiration. The correlation between the primary endpoint and thickening ratio through ROC analysis, and the cutoff value was 0.79 (AUCs 0.57, 95%CI 0.46–0.69). Subgroup analyses were also performed with ROC analysis. The cutoff value was also 0.79 when LSCEs as the endpoint. Lower than cutoff value indicates diaphragm dysfunction. We compared the impact of DD+ or DD− on endpoints by time-to-event analysis. In addition, we also analyzed the characteristics of participants between event group (who occurred target endpoints) and non-event group.

Continuous variables were expressed as means ± standard deviation or medians (25th, 75th percentiles). Categorical variables were expressed as counts(percentage). Continuous variables, according to the normality and homogeneity of variance, were compared by Wilcoxon test, Student’s t-test, or t’ test, respectively. Categorical variables were appropriately compared using chi-square test or Fisher’s exact test. The potential prognostic factors were screened one by one (α = 0.10) in Cox proportional hazard model, and then these screened out factors as covariates were taken into multivariate regression analysis by bootstrap. The results are shown as hazard ratios (HR) and 95% confident intervals (CI). All *p* values are two-tailed and difference denotes statistical significance at* P* < 0.05. Data were analyzed by the statistical software SAS 9.4 (SAS Institute Inc, USA), survival curves were drawn by R 4.0.3 (R Foundation for Statistical Computing, Vienna, Austria), histograms were drawn by GraphPad Prism 8.0.1(GraphPad Software Inc, USA).

## Results

### Participants

**Figure 1:**
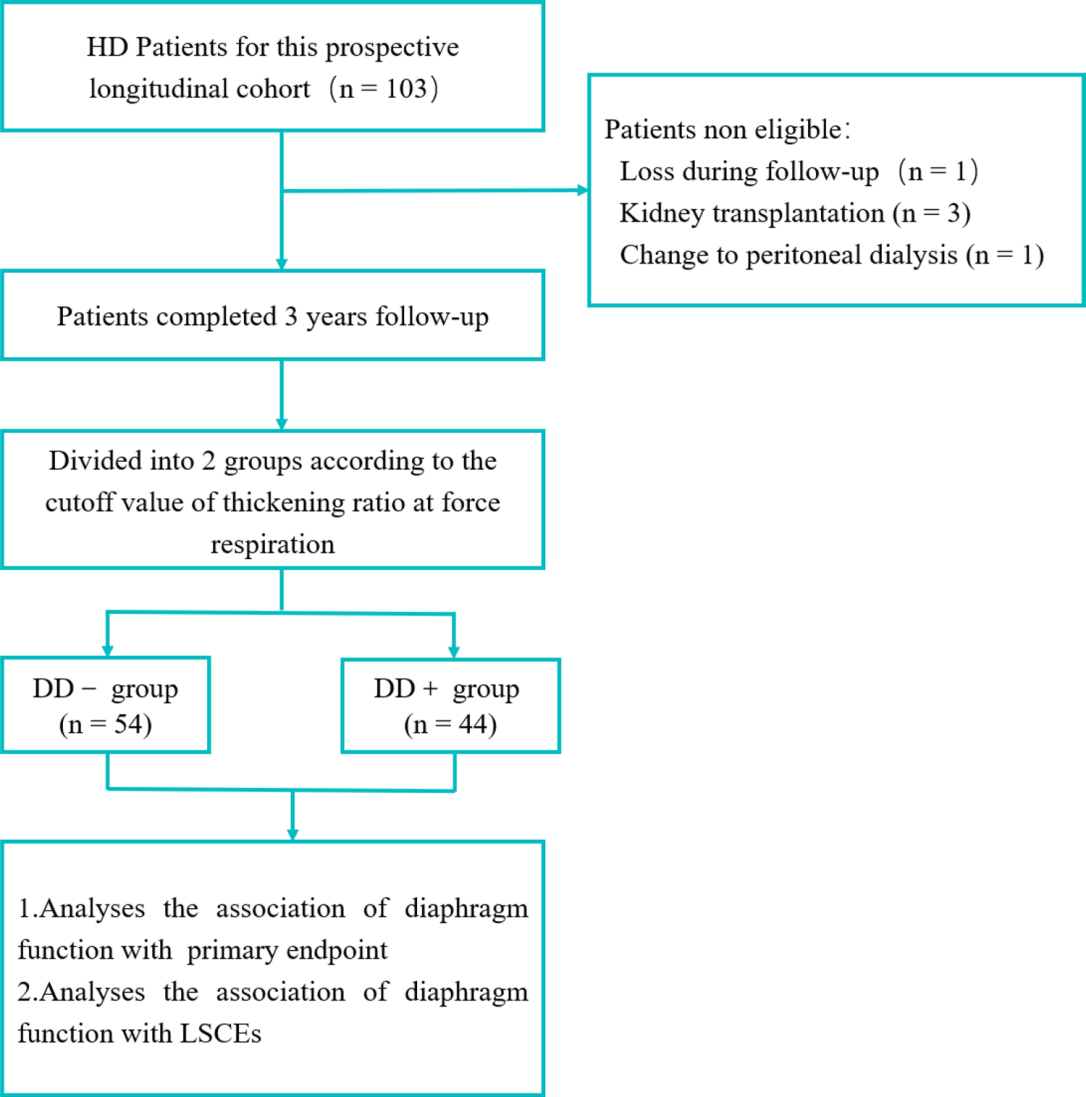
Flowchart of study participants.

The flowchart of this study is shown in Fig. 1. This cohort recruited a total of 103 patients. During the follow-up period, 1 participant lost follow-up because of changing residence to another province and altering his mobile phone number; 3 participants received kidney transplantation; and 1 participant changed to peritoneal dialysis. At the end of this study, 98 patients completed the scheduled follow-up data collection, including 57 men (58.16%). 81 of the 98 participants (82.65%) had finished 36 months follow-up.

### Baseline clinical characteristics of participants

**Figure 2:**
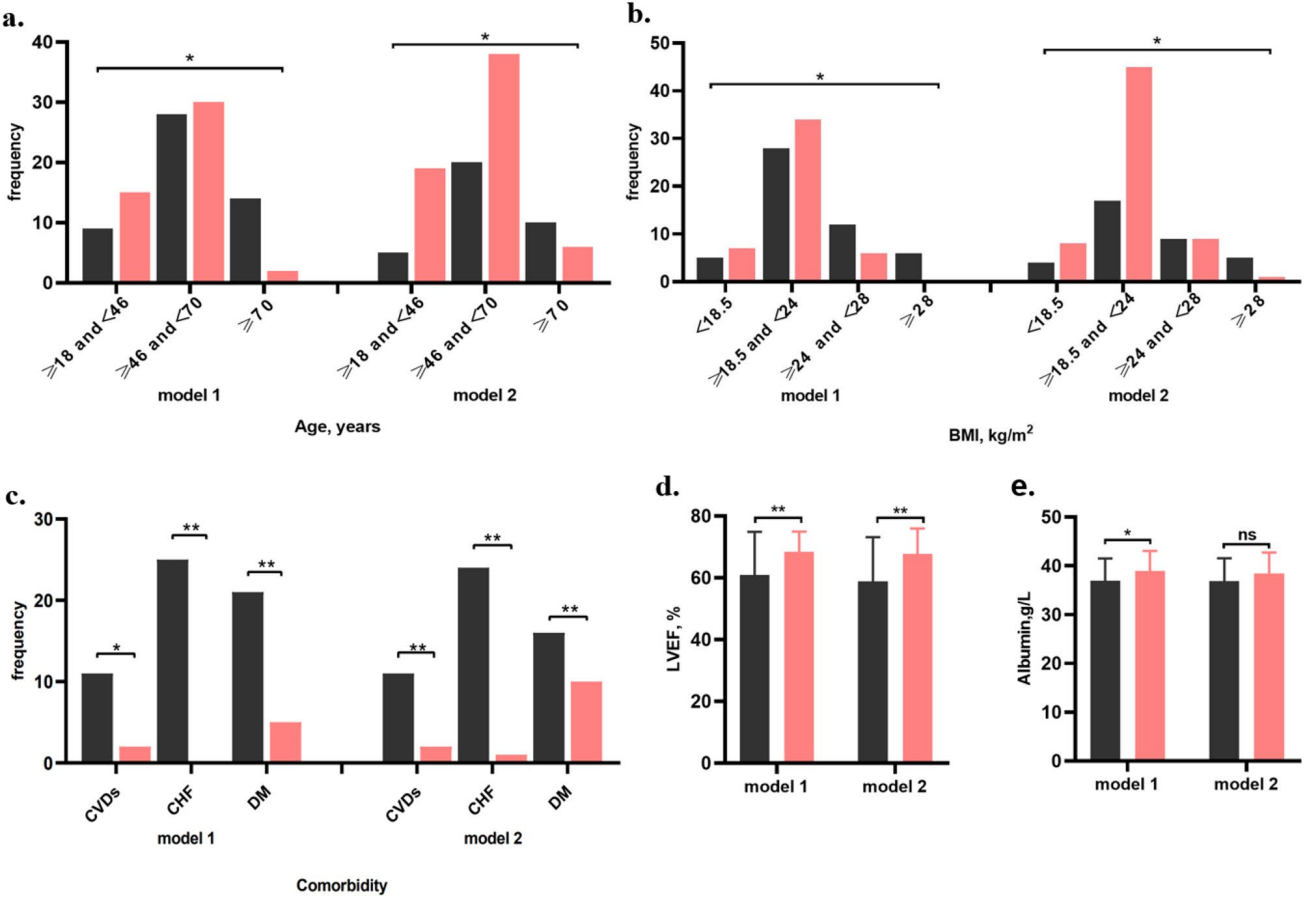
The differences of age (**a**), BMI (**b**), comorbidity (**c**), LVEF (**d**), serum levels of albumin (**e**) between event group and non-event group. BMI: body mass index; CVDs: cardiovascular diseases; CHF: chronic heart failure; DM: diabetes mellitus; LVEF: left ventricular ejection fraction; black column: event group; red column: non-event group; model 1: overall clinical events as the endpoints; model 2: LSCEs as the endpoints; ns: no significance; *p < 0.05; **p < 0.01.

Univariate analysis revealed only the serum level of hemoglobin was significantly higher in DD+ group than DD− group (*p* < 0.05), while other indexes of laboratory test and demography characteristics were not significantly different between DD+ group and DD− group (Table 1). Compared with the non-event group, the patients of event group had several traditional risk factors of CV diseases: older (more patients of age ≥ 70 years old and fewer of age < 45 years old), more comorbidity (CV diseases, chronic heart failure and diabetes mellitus), more overweight and obese patients. Moreover, patients in event group had lower LVEF (all *p* < 0.05, Fig. 2, Tables S1 and S2).

**Table 1:** The baseline clinical characteristics and incidents of all participants according to the diaphragm function.

Variables	DD− group (n = 54)	DD+ group (n = 44)	*p *value
**Age, year n (%)**			0.302
≥ 18 and < 45	14 (25.93)	10 (22.73)	
≥ 45 and < 70	34 (62.96)	24 (54.55)	
≥ 70	6 (11.11)	10 (22.73)	
Male, n (%)	34 (62.96)	23 (52.27)	0.286
**BMI, kg/m**^**2**^			0.228
< 18.5	5 (9.26)	7 (15.91)	
≥ 18.5 and < 24	39 (72.22)	23 (52.27)	
≥ 24 and < 28	8 (14.81)	10 (22.73)	
≥ 28	2 (3.70)	4 (9.09)	
Smoking, n (%)	22 (40.74)	17 (38.64)	0.832
**Complications**			
Hypertension, n (%)	52 (96.30)	40 (90.91)	0.404
CVDs, n (%)	4 (7.41)	9 (20.45)	0.058
CHF, n (%)	12 (22.22)	13 (29.55)	0.408
Diabetes mellitus, n (%)	12 (22.22)	14 (31.82)	0.285
**Combined drugs**			
ACEIs or ARBs, n (%)	20 (37.04)	18 (40.91)	0.696
*β*-blockers, n (%)	29 (53.70)	17 (38.64)	0.137
Statins, n (%)	4 (7.41)	0 (0.00)	0.125
Antiplatelet drugs, n (%)	13 (24.07)	15 (34.09)	0.275
**Laboratory test**			
Hemoglobin, g/L	98.65 ± 19.32	111.41 ± 20.62	0.002
Albumin, g/L	37.48 ± 4.25	38.44 ± 4.68	0.288
FBG, mmol/L	5.54 (4.60–7.43)	5.96 (4.74–7.91)	0.326
LVEF, %	67.00 (60.00–73.00)	67.5 (60.50–71.50)	0.977
TC, mmol/L	3.83 ± 0.97	3.81 ± 1.15	0.890
LDLC, mmol/L	2.08 (1.68–2.51)	2.16 (1.49–2.84)	0.974
**Parameters of diaphragm**			
Tdi_VT_, cm	0.28 (0.23–0.33)	0.26 (0.21–0.30)	0.131
Tdi_FRC_, cm	0.21 (0.17–0.25)	0.21 (0.17–0.25)	0.828
ΔTdi at eupnea, cm	0.07 (0.04–0.12)	0.04 (0.02–0.07)	< 0.001
Tdi_TLC_, cm	0.45 (0.35–0.52)	0.30 (0.26–0.37)	< 0.001
Tdi_RV_, cm	0.19 ± 0.06	0.22 ± 0.07	0.054
ΔTdi at forced respiration, cm	0.24 (0.18–0.31)	0.10 (0.07–0.12)	< 0.001
Thickening ratio at forced respiration	1.28 (0.99–1.63)	0.49 (0.36–0.57)	< 0.001
DE at eupnea, cm	2.73 (2.21–3.30)	2.32 (1.85–3.24)	0.265
Velocity at eupnea, cm	2.46 (1.94–2.92)	2.40 (1.84–3.22)	0.687
DE at forced respiration, cm	5.59 ± 2.00	4.89 ± 2.09	0.095
Velocity at forced respiration, cm/s	3.88 (2.69–4.83)	3.07 (2.23–4.59)	0.173
**Survival time, month**	26.74 ± 12.59	22.02 ± 12.98	0.046
**Incidents**			
Primary endpoint, n (%)	23 (42.59)	28 (63.64)	0.038
MACEs, n (%)	14 (25.93)	14 (31.82)	0.521
MACEs+, n (%)	6 (11.11)	6 (13.64)	0.705
LSCEs, n (%)	14 (25.93)	21 (47.73)	0.025

BMI, body mass index; CVDs, cardiovascular diseases; CHF, chronic heart failure; FBG, fasting blood glucose; LVEF, left ventricular ejection fraction; TC, total cholesterol; LDLC, low density lipoprotein cholesterol; ACEIs, angiotensin converting enzyme inhibitors; ARBs, angiotensin receptor blockers; DE, diaphragm excursion; MACEs, major adverse CV events; MACEs+, other major adverse CV events; LSCEs, less serious CV events; ΔTdi at eupnea, Tdi_VT_ − Tdi_FRC_; ΔTdi at force respiration, Tdi_TLC_ − Tdi_RV_; thickening ratio at forced respiration, (Tdi_TLC_ − Tdi_RV_) ∕ Tdi_RV_.

### Ultrasound-assessed baseline parameters of diaphragm

Tdi_TLC_, ΔTdi at eupnea, ΔTdi at forced respiration and thickening ratio at forced respiration were significantly lower in DD+ group (all *p* < 0.001, Table 1), compared to DD− group. DE at force respiration (*p* = 0.095, Table 1) was lower in DD+ group with a trend toward statistical significance. However, compared to non-event group, only DE at force respiration were significantly lower in the primary endpoint event group (*p* < 0.05, Table S1). Several parameters of diaphragm thickness were significantly thicker in the LSCEs’ event group: Tdi_VT_ (*p* = 0.026), Tdi_FRC_ (*p* = 0.040), and Tdi_RV_ (*p* = 0.010) (Table S2).

### Primary endpoint

**Figure 3:**
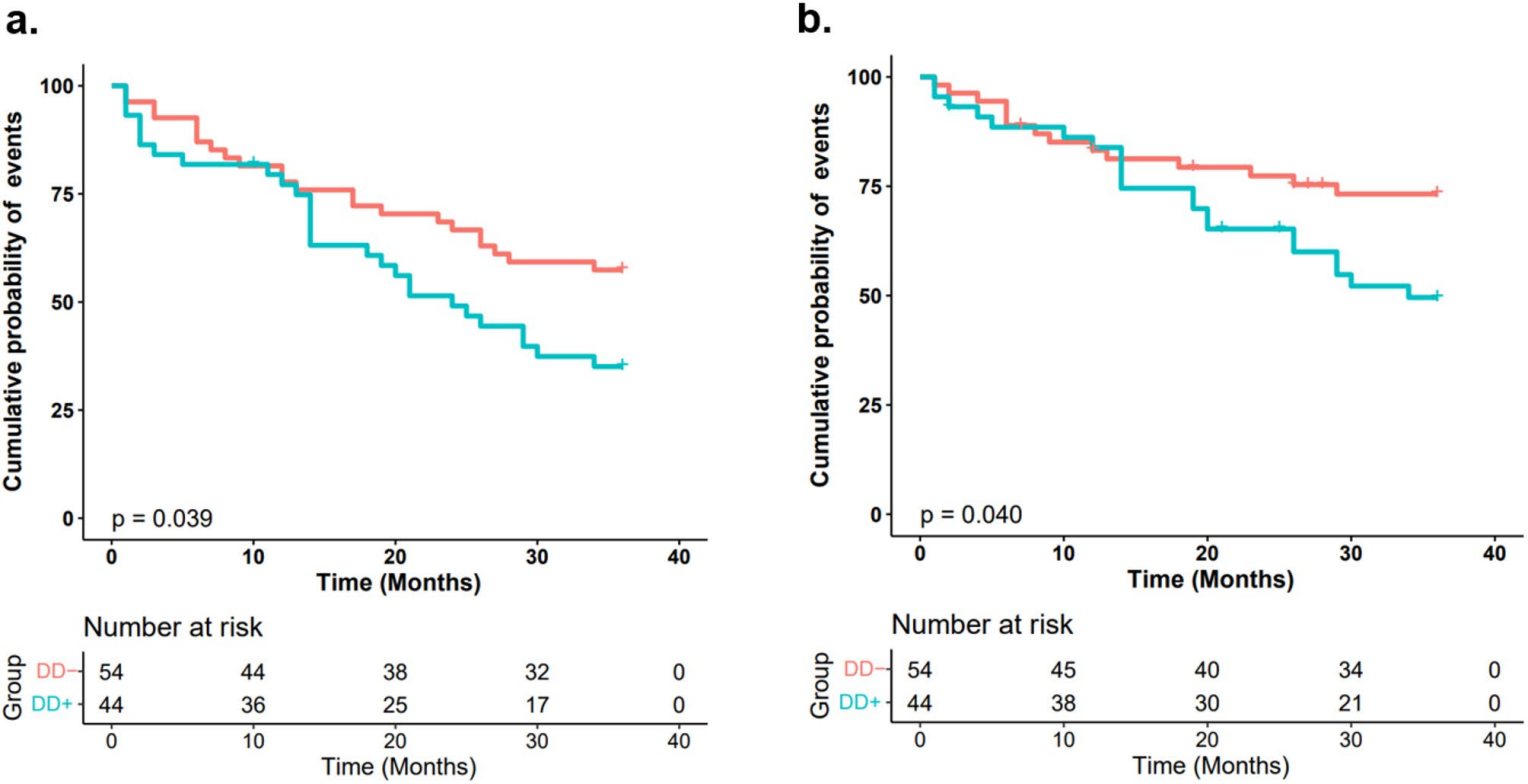
Kaplan–Meier analysis by log-rank test. (**a**) the endpoint was a composite of the first nonfatal CV event and all-cause mortality; (**b**) the endpoint was LSCEs; DD+: diaphragm dysfunction (blue line); DD−: normal diaphragm function (red line).

During the follow-up period, 17 of the 98 participants (17.35%) died, and 10 (10.20%) died of CV events. Among the other 7 (7.14%) patients, 2 (2.04%) died of tumor, 2 (2.04%) died due to delayed dialysis during COVID-19 pandemic, 2 (2.04%) died of septic shock, 1 (1.02%) died of gastrointestinal and urinary tract bleeding. Compared to the DD− group, the DD+ group had significantly shorter survival time (22.02 ± 12.98 months *vs.* 26.74 ± 12.59 months, *p* = 0.046). 28 of the 44 patients(63.64%) in DD+ group and 23 of the 54 patients (42.59%) in DD− group had at least one nonfatal CV event or all-cause mortality (*p* = 0.038,Table 1). Cumulative probability of the primary endpoint in DD+ group was significantly higher than DD− group (*p* = 0.039, Fig. 3a). After adjusting confounding factors, the DD+ group possessed significantly heightened risk of the composite of nonfatal CV events and all-cause mortality (HR 1.59, 95%CI 1.54–1.63, Table 2).

**Table 2:** Univariate analysis by Cox proportional hazard analysis and multivariate analysis by bootstrap for primary endpoint.

Factors	Univariate analysis				Multivariate analysis			
	SE	WaldX2	*p* value	HR(95%CI)	SE	*Z*-value	*p* value	HR(95%CI)
**Age**								
≥ 45and < 70	0.38	0.76	0.283	1.40 (0.66–2.96)	0.03	4.86	< 0.001	1.15 (1.09–1.21)
≥ 70	0.44	10.87	0.001	4.23 (1.79–9.96)	0.04	35.31	< 0.001	3.45 (3.22–3.70)
Male	0.29	1.06	0.303	1.35 (0.76–2.38)				
**BMI**								
< 18.5	0.49	0.08	0.779	0.87 (0.34–2.26)	0.05	− 15.28	< 0.001	0.47 (0.43–0.52)
≥ 24 and < 28	0. 35	2.67	0.102	1.76 (0.89–3.47)	0.02	15.20	< 0.001	1.33 (1.28–1.38)
≥ 28	0.46	7.53	0.006	3.55 (1.44–8.76)	0.03	15.48	< 0.001	1.50 (1.42–1.58)
Hypertension (yes)	0.72	0.46	0.497	1.63 (0.40–6.72)				
CVDs (yes)	0.35	11.59	< 0.001	3.34 (1.67–6.69)	0.02	19.84	< 0.001	2.62 (2.59–2.65)
CHF (yes)	0.33	46.45	< 0.001	9.17 (4.85–17.35)	0.02	92.10	< 0.001	7.48 (7.16–7.81)
Diabetes mellitus (yes)	0.29	10.93	< 0.001	2.59 (1.47–4.54)	0.02	6.19	< 0.001	1.13 (1.09–1.17)
Smoking(yes)	0.28	3.21	0.073	1.66 (0.95–2.87)	0.01	11.29	< 0.001	1.18 (1.14–1.21)
Hemoglobin	0.01	0.06	0.804	1.00 (0.99–1.02)				
Albumin	0.03	4.74	0.030	0.93 (0.87–0.99)	0.00	− 17.55	< 0.001	0.97 (0.97–0.98)
FBG	0.04	8.06	0.005	1.13 (1.04–1.23)	0.00	− 5.85	< 0.001	0.99 (0.98–0.99)
LVEF	0.01	15.65	< 0.001	0.96 (0.94–0.98)	0.00	− 36.99	< 0.001	0.97 (0.97–0.98)
DD+	0.28	4.04	0.044	1.76 (1.01–3.07)	0.01	32.01	< 0.001	1.59 (1.54–1.63)
TC	0.13	1.88	0.170	0.84 (0.65–1.08)				
LDLC	0.19	3.01	0.083	0.73 (0.50–1.04)	0.01	− 24.79	< 0.001	0.82 (0.81–0.84)
ACEIs or ARBs(yes)	0.29	0.03	0.857	0.95 (0.54–1.68)				
*β*-blockers(yes)	0.28	1.13	0.288	0.74 (0.42–1.29)				
Statins(yes)	0.72	0.01	0.926	1.07 (0.26–4.40)				
Antiplatelet drugs(yes)	0.29	10.10	0.002	2.52 (1.42–4.45)	0.02	30.86	< 0.001	1.75 (1.69–1.82)

SE, standard error; HR, hazard ratio; CI, confidence interval; BMI, body mass index; CVDs, cardiovascular diseases; CHF, chronic heart failure; FBG, fasting blood glucose; LVEF, left ventricular ejection fraction; DD+, diaphragm dysfunction; TC, total cholesterol; LDLC, low density lipoprotein cholesterol; ACEIs, angiotensin converting enzyme inhibitors; ARBs, angiotensin receptor blockers.

### Secondary endpoints

There were no significant differences in the occurrences of MACEs or MACEs+ between DD− group and DD+ group (both *p* > 0.05, Table 1). While the incidences of LSCEs were significant higher in DD+ group than DD− group (*p* = 0.025, Table 1). 21 of the 35 occurrences of LSCEs (60%) were rehospitalization for heart failure. Cumulative probability of LSCEs in DD+ group was significant higher, compared to DD− group (*p* = 0.040, Fig. 3b). After adjusting confounding factors, diaphragm dysfunction was also a risk factor of LSCEs (HR 1.47, 95%CI 1.40–1.55, Table 3) in HD patients. However, the similar adverse effect of diaphragm dysfunction was not found when MACEs or MACEs+ as endpoint (Tables S3, S4 and Figure S1).

**Table 3:** Univariate analysis by Cox proportional hazard analysis and multivariate analysis by bootstrap for LSCEs as the endpoint.

Factors	Univariate analysis				Multivariate analysis			
	SE	WaldX2	*p* value	HR(95%CI)	SE	*Z*-value	*p* value	HR(95%CI)
**Age**								
≥ 45and < 70	0.50	1.73	0.189	1.93 (0.72–5.15)	0.05	10.98	< 0.001	1.79 (1.62–1.99)
≥ 70	0.56	9.28	0.002	5.47 (1.83–16.34)	0.07	24.65	< 0.001	5.01 (4.41–5.69)
Male	0.35	0.38	0.536	1.24 (0.63–2.47)				
**BMI**								
< 18.5	0.56	0.12	0.727	1.22 (0.41–3.61)	0.06	0.76	0.225	1.05 (0.93–1.19)
≥ 24 and < 28	0.41	2.59	0.108	1.94 (0.87–4.36)	0.04	13.41	< 0.001	1.67 (1.55–1.80)
≥ 28	0.52	9.21	0.002	4.90 (1.76–13.66)	0.05	10.62	< 0.001	1.72 (1.56–1.90)
Hypertension(yes)	1.01	0.85	0.356	2.55 (0.35–18.63)				
CVDs (yes)	0.38	20.00	< 0.001	5.45 (2.59–11.44)	0.03	10.15	< 0.001	1.38 (1.30–1.47)
CHF (yes)	0.43	55.91	< 0.001	24.24 (10.51–55.90)	0.06	58.44	< 0.001	31.39 (27.96–35.24)
Diabetes mellitus (yes)	0.34	10.76	0.001	3.07 (1.57–5.99)	0.04	6.08	< 0.001	1.28 (1.18–1.38)
Smoking(yes)	0.34	2.31	0.129	1.67 (0.86–3.25)				
Hemoglobin	0.01	0.26	0.608	1.00 (0.98–1.01)				
Albumin	0.04	4.60	0.032	0.92 (0.85–0.99)	0.00	− 19.99	< 0.001	0.96 (0.95–0.96)
FBG	0.06	1.80	0.180	1.08 (0.97–1.20)				
LVEF	0.01	19.43	< 0.001	0.95 (0.93–0.97)	0.00	− 48.67	< 0.001	0.96 (0.95–0.96)
DD+	0.35	3.98	0.046	1.99 (1.01–3.92)	0.02	15.73	< 0.001	1.47 (1.40–1.55)
TC	0.16	0.60	0.440	0.89 (0.65–1.21)				
LDLC	0.23	1.94	0.163	0.73 (0.47–1.14)				
ACEIs or ARBs(yes)	0.35	0.03	0.865	1.06 (0.54–2.09)				
*β*-blockers(yes)	0.35	1.31	0.252	0.67 (0.34–1.33)				
Statins(yes)	1.02	0.04	0.839	0.81 (0.11–5.95)				
Antiplatelet drugs(yes)	0.34	6.61	0.010	2.42 (1.23–4.73)	0.02	− 4.32	< 0.001	0.91 (0.87–0.95)

SE, standard error; HR, hazard ratio; CI, confidence interval; BMI, body mass index; CVDs, cardiovascular diseases; CHF, chronic heart failure; FBG, fasting blood glucose; LVEF, left ventricular ejection fraction; DD+, diaphragm dysfunction; TC, total cholesterol; LDLC, low density lipoprotein cholesterol; ACEIs, angiotensin converting enzyme inhibitors; ARBs, angiotensin receptor blockers.

## Discussion

This is the first prospective longitudinal cohort study about examining the association between diaphragm dysfunction and clinical outcomes in HD patients. The main finding of this study is that diaphragm dysfunction, identified by ultrasound-assessed thickening ratio at forced respiration, predicts the clinical outcomes in HD patients. HD patients with diaphragm dysfunction increases future occurrence of composite of nonfatal CV events and mortality, and particularly LSCEs, independent of other confounding factors, such as age, BMI, smoking, concomitant diseases (including hypertension, CV diseases, chronic heart failure, and diabetes mellitus), and serum level of LDLC.

Diaphragmatic movement not only affects respiratory, but also cardiac output. As respiratory work reduced, stroke volume and cardiac output decreased^29^. In this study, diaphragm dysfunction was particularly associated with LSCEs, majority of which was heart failure readmission. Inspiratory muscle dysfunction had been found to correlate with CHF and as an independent predictor of prognosis in CHF patients^16,30^. Decreased caudal displacement of diaphragm could directly reduce lung volume and negative intrathoracic pressure, leading to imbalance of ventilation and perfusion ratio of lung, sympathetic activation, and decreased cardiac output^31–33^. During this pathophysiology, heart failure, arrythmia and myocardial ischemia are all apt to occur. Recently, dyspnea which cannot be explained by cardiopulmonary disease was named as “residual exertional dyspnea”, and one of its possible causes was diaphragm dysfunction^34^. In addition to nutritional intervention, simple resistance training can ameliorate diaphragm dysfunction^35^. However, diaphragm dysfunction has not attached enough attention of clinicians.

There is no association of diaphragm dysfunction with MACEs or MACEs+ in our cohort. The major underlying pathology of cardiovascular diseases included in MACEs and MACEs+ is atherosclerosis^36,37^. CKD expedites atherosclerosis by augmenting inflammation, dyslipidemia, vascular calcification, immune disorders and other mechanisms^38^. Skeleton muscle can communicate with heart by molecular factors^39^. However, the slow progression of atherosclerotic plaque to occur CV events often requires a relatively long time, and clinicians generally have attached importance to secondary prevention of atherosclerotic diseases. Prolonged follow-up time may be needed to explore the underlying relationships of diaphragm function with MACEs and MACEs+.

CKD is often accompanied by skeletal muscle wasting, characterized by loss of muscle mass, reduced muscle strength and function. ΔTdi at eupnea, ΔTdi and thickening ratio at forced respiration are significantly lower in DD+ group (Table 1), reminds that the changes in diaphragm thickness are reduced in both maneuvers. This suggests that contraction and expansion of diaphragm have been impaired in DD+ group. Diaphragm thickness was positively correlated with diaphragm contractile activity^40^, and Tdi_TLC_ below 4.0 mm was defined as impaired diaphragm function in HF patients^41^. In this study, Tdi_TLC_ is significantly thinner in DD+ group than DD− group (Table 1). However, there were no difference in endpoints when Tdi_TLC_ was used to differentiated DD+ group and DD− group (Data not shown). The reason of which may be that muscle mass and muscle function are incongruent in CKD patients. Impaired muscle function in uremic milieu predated muscle atrophy^42,43^. Compared Tdi of event group with non-event group, several parameters are even thicker (Table S2). The reason of which may be the potential compensatory mechanism to increase diaphragm contraction^44^.

Except for ultrasonography, the following means—chest radiographs, fluoroscopy, sniff test, pulmonary-function tests, maximal static inspiratory pressure, sniff nasal inspiratory pressure, transdiaphragmatic pressure, electromyography, have been used for assessing diaphragm function^45^. Compared with these examinations, ultrasonography is a noninvasive, nonradiative, reproducible and costless technique. Ultrasound can measure the length, thickness, motion and strain of diaphragm^24,46,47^. Although it can’t directly provide the diaphragm muscle strength, the application prospect is encouraging.

Strength of this study reveals HD patients with lower thickening ratio at forced respiration of diaphragm have a significant higher probability of adverse clinical events. As the follow-up time went on, the difference became more obvious. It should be emphasized that the majority of patients included in our cohort had good nutritional status [only 12 of 98 (12.24%) patients were underweight and the average serum level of albumin was > 35 g/L], however, a part of participants had appeared a relatively decline in diaphragm function evaluated by thickening ratio at forced respiration. Thus, ultrasound-assessed thickening ratio at forced respiration is a potential indicator of early identification of diaphragm dysfunction in HD patients.

We should declare several limitations of our study. First, expanded sample size was needed to find the appropriate cutoff value of thickening ratio at forced respiration, having ideal sensitivity and specificity, to differentiate DD− and DD+. Second, statins and anti-platelet drugs were not used by all patients at risk of cardiovascular diseases, and the type and dosage of similar drug varied. This would bias the incidents of MACEs and MACEs+. Third, it did not perform pulmonary function tests and diaphragm ultrasound at the same time, so this study didn’t show the relationship between diaphragm function and pulmonary function in HD patients directly. Fourth, correlation analysis does not imply causation, longitudinal trial with specific intervention for improving diaphragm dysfunction to observe the effect on prognosis would be necessary.

## Conclusion

Ultrasonography is an ideal tool and easy to be operated for evaluating diaphragm function. Low thickening ratio at forced respiration is an early indicator of diaphragm dysfunction and is associated with adverse clinical outcomes in HD patients. These findings suggests that routine screening for diaphragm dysfunction is advisable in HD patients and intervention of diaphragm dysfunction is expected to be a novel strategy for improving the clinical outcomes in HD patients.

## Supplementary Information

Supplementary Information.

## Data Availability

The datasets generated and/or analyzed during the current study are available from the corresponding author on reasonable request.
