# Supplementary material for: Ultrasound-assessed diaphragm dysfunction predicts clinical outcomes in hemodialysis patients
Source: Sci Rep. 2022 Oct 3;12:16550. doi: 10.1038/s41598-022-20450-x (PMC9529158; doi:10.1038/s41598-022-20450-x)
Supplement: Supplementary file 1 — Supplementary Information. [file 41598_2022_20450_MOESM1_ESM.docx]

**Ultrasound-assessed Diaphragm Dysfunction Predicts Clinical Outcomes in Hemodialysis Patients**

Jing Zheng^1.2^, Qing Yin^1^, Shi-yuan Wang^3^, Ying-Yan Wang^4^, Jing-jie Xiao^5^, Tao-tao Tang^1^, Wei-jie Ni^1^, Li-qun Ren^2^, Hong Liu^1^, Xiao-liang Zhang^1^, Bi-Cheng Liu^1*^& Bin Wang^1*^

^1^Institute of Nephrology, ^2^Department of Gerontology, ^4^Department of Ultrasound Medicine, Zhong Da Hospital, Southeast University School of Medicine, Nanjing, Jiangsu, China.

^3^Department epidemiology & health statistics, Southeast University, Nanjing, Jiangsu, China.

^5^Covenant Health Palliative Institute, Edmonton, Alberta, Canada.

Running title: Diaphragm Dysfunction Increases Clinical Events in HD Patients

*Correspondence: Bi-Cheng Liu, MD, PhD & Bin Wang, MD, PhD

Institute of Nephrology, Zhong Da Hospital, Southeast University School of Medicine, Nanjing, Jiangsu, China.

Address: No. 87, Dingjiaqiao Road, Gulou District, Nanjing, Jiangsu Province, China.

E-mail address: [wangbinhewei@126.com &](mailto:wangbinhewei@126.com%20&) liubc64@163.com

Tel: 0086 25 83262422

Fax: 0086 25 83262422

**TableS1** Clinical characteristics of all participants according to whether the primary endpoint occurred

| **Variables** | **Non-event group** | **Event group** | ***P* value** |
| --- | --- | --- | --- |
|  | **(n=47)** | **(n=51)** |  |
| Age, year, n (%) |  |  | ***0.005*** |
| ≥18 and ＜45 | 15(34.04) | 9(17.65) |  |
| ≥45 and ＜70 | 30(61.70) | 28(54.90) |  |
| ≥70 | 2(4.26) | 14(27.45) |  |
| Male, n (%) | 25(53.19) | 32(62.75) | 0.338 |
| BMI, kg/m^2^ |  |  | ***0.028*** |
| ＜18.5 | 7(14.89) | 5(9.80) |  |
| ≥18.5 and ＜24 | 34(72.34) | 28(54.90) |  |
| ≥24 and ＜28 | 6(12.77) | 12(23.53) |  |
| ≥28 | 0(0.00) | 6(11.76) |  |
| Smoking, n (%) | 15(31.91) | 24(47.06) | 0.126 |
| **Comorbidity** |  |  |  |
| Hypertension, n (%) | 43(91.49) | 49(96.08) | 0.423 |
| CVDs, n (%) | 2(4.26) | 11(21.57) | ***0.012*** |
| CHF, n (%) | 0(0.00) | 25(49.02) | ***<0.001*** |
| DM, n (%) | 5(10.64) | 21(41.18) | ***<0.001*** |
| **Drugs** |  |  |  |
| ACEIs or ARBs, n (%) | 19(40.43) | 19(37.25) | 0.748 |
| β-blockers, n (%) | 25(53.19) | 21(41.18) | 0.234 |
| Statins, n (%) | 2(4.26) | 2(3.92) | 1.000 |
| Antiplatelet drugs, n (%) | 8(17.02) | 20(39.22) | ***0.015*** |
| **Laboratory test** |  |  |  |
| Hemoglobin, g/L | 103.72±18.20 | 104.98±23.13 | 0.767 |
| Albumin, g/L | 38.93±4.14 | 36.97±4.56 | ***0.028*** |
| FBG, mmol/L | 5.04(4.49～6.42) | 6.53(4.88～8.12) | ***0.009*** |
| LVEF, % | 68.40±6.54 | 60.98±13.91 | ***0.001*** |
| TC, mmol/L | 4.01±1.10 | 3.65±0.98 | 0.083 |
| LDLC, mmol/L | 2.32±0.83 | 2.02±0.71 | 0.058 |
| **Parameters of diaphragm** |  |  |  |
| TdiVT, cm | 0.26(0.19～0.31) | 0.27(0.23～0.33) | 0.174 |
| TdiFRC, cm | 0.20(0.15～0.25) | 0.22(0.19～0.26) | 0.062 |
| ΔTdi at eupnea, cm | 0.06(0.03～0.08) | 0.06(0.02～0.09) | 0.741 |
| TdiTLC, cm | 0.38(0.28～0.49) | 0.37(0.29～0.48) | 0.938 |
| TdiRV, cm | 0.20±0.06 | 0.21±0.07 | 0.239 |
| ΔTdi at force respiration, cm | 0.18(0.11～0.27) | 0.14(0.10～0.26) | 0.546 |
| Thickening ratio at forced respiration | 1.03±0.57 | 0.90±0.54 | 0.249 |
| DE at eupnea, cm | 2.57(2.11～3.45) | 2.67(1.86～3.28) | 0.486 |
| Velocity at eupnea, cm/s | 2.49±0.83 | 2.63±0.97 | 0.461 |
| DE at forced respiration, cm | 5.21(4.12～7.33) | 4.52(3.17～5.91) | ***0.017*** |
| Velocity at forced respiration, cm/s | 3.63(2.38～4.73) | 3.53(2.48～4.83) | 0.952 |
| **Survival time, month** | 35.45±3.79 | 14.65±9.95 | ***<0.001*** |

BMI: body mass index; CVDs: cardiovascular diseases; CHF: chronic heart failure; DM: diabetes mellitus; FBG: fasting blood glucose; LVEF: left ventricular ejection fraction; TC: total cholesterol; LDLC: low density lipoprotein cholesterol; ACEIs: angiotensin converting enzyme inhibitors; ARBs: angiotensin receptor blockers; DE: diaphragm excursion；ΔTdi at eupnea: Tdi_VT_ −Tdi_FRC_；ΔTdi at force respiration: Tdi_TLC_−Tdi_RV_; Thickening ratio at forced respiration: (Tdi_TLC_ − Tdi_RV_) ∕ Tdi_RV_.

**TableS2** Clinical characteristics of all participants according to whether the LSCEs occurred

| **Variables** | **Non-event group** | **Event group** | ***P* value** |
| --- | --- | --- | --- |
|  | **(n=63)** | **(n=35)** |  |
| Age, year, n(%) |  |  | ***0.025*** |
| ≥18 and ＜45 | 19(30.16) | 5(14.29) |  |
| ≥45 and ＜70 | 38(60.32) | 20(57.14) |  |
| ≥70 | 6(9.52) | 10(28.57) |  |
| Male, n(%) | 35(55.56) | 22(62.86) | 0.483 |
| BMI, kg/m^2^ |  |  | ***0.025*** |
| ＜18.5 | 8(12.70) | 4(11.43) |  |
| ≥18.5 and ＜24 | 45(71.43) | 17(48.57) |  |
| ≥24 and ＜28 | 9(14.29) | 9(25.71) |  |
| ≥28 | 1(1.59) | 5(14.29) |  |
| Smoking, n(%) | 22(34.92) | 17(48.57) | 0.186 |
| **Complications** |  |  |  |
| Hypertension, n(%) | 58(92.06) | 34(97.14) | 0.416 |
| CVDs, n(%) | 2(3.17) | 11(31.43) | ***<0.001*** |
| CHF, n(%) | 1(1.59) | 24(68.57) | ***<0.001*** |
| DM, n(%) | 10(15.87) | 16(45.71) | ***0.001*** |
| **Combined drugs** |  |  |  |
| ACEIs or ARBs, n(%) | 24(38.10) | 14(40.00) | 0.853 |
| *β*-blockers, n(%) | 32(50.79) | 14(40.00) | 0.305 |
| Statins, n(%) | 3(4.76) | 1(2.86) | 1.000 |
| Antiplatelet drugs, n(%) | 13(20.63) | 15(42.86) | 0.020 |
| **Laboratory test** |  |  |  |
| Hemoglobin, g/L | 104.51±20.09 | 104.14±22.35 | 0.934 |
| Albumin, g/L | 38.47±4.25 | 36.90±4.68 | 0.093 |
| FBG, mmol/L | 5.26(4.51～7.48) | 6.53(4.88～8.06) | 0.077 |
| LVEF, % | 69.00(63.00～73.00) | 62.00(54.00～70.00) | ***0.002*** |
| TC, mmol/L | 3.88±1.15 | 3.71±0.85 | 0.393 |
| LDLC, mmol/L | 2.24±0.84 | 2.03±0.64 | 0.206 |
| **Parameters of diaphragm** |  |  |  |
| TdiVT, cm | 0.25(0.22～0.31) | 0.29(0.24～0.36) | ***0.026*** |
| TdiFRC, cm | 0.21±0.07 | 0.24±0.08 | ***0.040*** |
| ΔTdi at eupnea, cm | 0.06(0.03～0.09) | 0.06(0.03～0.11) | 0.714 |
| TdiTLC, cm | 0.35(0.28～0.47) | 0.38(0.29～0.50) | 0.328 |
| TdiRV, cm | 0.19±0.06 | 0.23±0.07 | ***0.010*** |
| ΔTdi at forced respiration, cm | 0.17(0.11～0.26) | 0.15(0.10～0.28) | 0.775 |
| Thickening ratio at forced respiration | 0.91(0.54～1.44) | 0.74(0.47～1.28) | 0.141 |
| DE at eupnea, cm | 2.51(2.01～3.28) | 2.73(1.50～3.47) | 0.941 |
| Velocity at eupnea, cm/s | 2.33(1.89～3.21) | 2.52(1.94～2.94) | 0.484 |
| DE at forced respiration, cm | 5.20(3.80～6.70) | 4.56(3.13～6.74) | 0.236 |
| Velocity at forced respiration, cm/s | 3.63(2.56～4.71) | 3.22(2.45～5.14) | 0.926 |
| **Survival time, month** | 33.27±7.19 | 14.46±9.89 | ***<0.001*** |

BMI: body mass index; CVDs: cardiovascular diseases; CHF: chronic heart failure; DM: diabetes mellitus; FBG: fasting blood glucose; LVEF: left ventricular ejection fraction; TC: total cholesterol; LDLC: low density lipoprotein cholesterol; ACEIs: angiotensin converting enzyme inhibitors; ARBs: angiotensin receptor blockers; DE: diaphragm excursion；ΔTdi at eupnea: Tdi_VT_ −Tdi_FRC_；ΔTdi at force respiration: Tdi_TLC_−Tdi_RV_; Thickening ratio at forced respiration: (Tdi_TLC_ − Tdi_RV_) ∕ Tdi_RV_.

**TableS3** Univariate analysis by Cox proportional hazard analysis and multivariate analysis by bootstrap for MACEs as the endpoint

| Factors | univariate analysis | | | |  | | multivariate analysis | | | |
| --- | --- | --- | --- | --- | --- | --- | --- | --- | --- | --- |
|  | SE | WaldX2 | *P*-value | HR（95%CI） | |  | SE | *Z*-value | *P*-value | HR（95%CI） |
| Age |  |  |  |  | |  |  |  |  |  |
| ≥45 and ＜70 | 0.76 | 1.95 | 0.163 | 2.89(0.65-12.79) | |  | 0.16 | 18.05 | <0.001 | 17.65(12.92-24.10) |
| ≥70 | 0.78 | 9.23 | 0.002 | 10.59(2.31-48.54) | |  | 0.16 | 25.52 | <0.001 | 64.93(47.12-89.45) |
| Male | 0.42 | 0.62 | 0.430 | 1.39(0.61-3.15) | |  |  |  |  |  |
| BMI |  |  |  |  | |  |  |  |  |  |
| ＜18.5 | 0.75 | 0.26 | 0.612 | 0.68(0.16-2.98) | |  |  |  |  |  |
| ≥24 and ＜28 | 0.52 | 0.20 | 0.652 | 1.26(0.46-3.47) | |  |  |  |  |  |
| ≥28 | 0.63 | 2.20 | 0.138 | 2.56(0.74-8.85) | |  |  |  |  |  |
| Hypertension (yes) | 0.74 | 0.16 | 0.688 | 0.74(0.18-3.16) | |  |  |  |  |  |
| CVDs (yes) | 0.47 | 4.19 | 0.041 | 2.61(1.04-5.18) | |  | 0.04 | 18.84 | <0.001 | 2.25(2.07-2.45) |
| CHF (yes) | 0.44 | 6.20 | 0.013 | 2.97(1.26-7.01) | |  | 0.03 | -7.41 | <0.001 | 0.80(0.76-0.85) |
| DM (yes) | 0.41 | 3.91 | 0.048 | 2.24(1.01-5.00) | |  | 0.03 | -12.50 | <0.001 | 0.71(0.68-0.75) |
| Smoking (yes) | 0.40 | 4.41 | 0.036 | 2.33(1.06-5.14) | |  | 0.03 | 14.54 | <0.001 | 1.44(1.38-1.52) |
| Hemoglobin | 0.01 | 0.01 | 0.916 | 1.00(0.98-1.02) | |  |  |  |  |  |
| Albumin | 0.05 | 3.86 | 0.050 | 0.92(0.84-1.00) | |  | 0.00 | -30.79 | <0.001 | 0.93(0.92-0.93) |
| FBG | 0.06 | 12.32 | <0.001 | 1.22(1.09-1.36) | |  | 0.00 | 47.85 | <0.001 | 1.24(1.23-1.25) |
| LVEF | 0.00 | 16.21 | <0.001 | 1.00(1.00-1.00) | |  | 0.00 | -49.60 | <0.001 | 1.00(1.00-1.00) |
| DD+ | 0.41 | 0.47 | 0.492 | 1.32(0.60-2.95) | |  |  |  |  |  |
| TC | 0.20 | 4.30 | 0.038 | 0.66(0.45-0.98) | |  | 0.03 | -17.53 | <0.001 | 0.56(0.52-0.60) |
| LDLC | 0.29 | 3.22 | 0.073 | 0.59(0.33-1.05) | |  | 0.05 | 12.60 | <0.001 | 1.77(1.62-1.94) |
| ACEIs or ARBs (yes) | 0.45 | 1.72 | 0.189 | 0.56(0.23-1.33) | |  |  |  |  |  |
| *β*-blockers (yes) | 0.42 | 1.44 | 0.231 | 0.61(0.27-1.37) | |  |  |  |  |  |
| Statins (yes) | 0.74 | 2.01 | 0.156 | 2.85(0.67-12.11) | |  |  |  |  |  |
| Antiplatelet drugs (yes) | 0.42 | 1.33 | 0.287 | 1.56(0.69-3.53) | |  |  |  |  |  |

BMI: body mass index; CVDs: cardiovascular diseases; CHF: chronic heart failure; DM: diabetes mellitus; FBG: fasting blood glucose; LVEF: left ventricular ejection fraction; DD: diaphragm dysfunction; TC: total cholesterol; LDLC: low density lipoprotein cholesterol; ACEIs: angiotensin converting enzyme inhibitors; ARBs: angiotensin receptor blockers.

**TableS4** Univariate analysis by Cox proportional hazard analysis and multivariate analysis by bootstrap for MACEs+ as the endpoint. In this model, multivariate analysis failed to estimate out results. So, this table only shows the results estimated by univariate analysis**.**

| Factors | univariate analysis | | | |
| --- | --- | --- | --- | --- |
|  | SE | WaldX2 | *P*-value | HR（95%CI） |
| Age |  |  |  |  |
| ≥45 and ＜70 | 0.71 | 0.02 | 0.894 | 0.91(0.23-3.64) |
| ≥70 | 0.82 | 0.49 | 0.483 | 1.78(0.36-8.84) |
| Male | 0.67 | 1.48 | 0.223 | 2.25(0.61-8.32) |
| BMI |  |  |  |  |
| ＜18.5 | 1.12 | 0.04 | 0.847 | 1.24(0.14-11.11) |
| ≥24 and ＜28 | 0.63 | 9.45 | 0.002 | 6.88(2.01-23.53) |
| ≥28 | 2276.41 | 0.00 | 0.995 | <0.01(<0.01-.) |
| Hypertension (yes) | 1.04 | 0.20 | 0.655 | 0.63(0.08-4.86) |
| CVDs (yes) | 0.62 | 5.31 | 0.021 | 4.14(1.24-13.85) |
| CHF (yes) | 0.59 | 2.02 | 0.155 | 2.30(0.73-7.25) |
| DM (yes) | 0.58 | 4.02 | 0.045 | 3.19(1.03-9.90) |
| Smoking (yes) | 0.61 | 3.89 | 0.049 | 3.35(1.01-11.12) |
| Hemoglobin | 0.01 | 2.17 | 0.141 | 1.02(0.99-1.05) |
| Albumin | 0.06 | 0.00 | 0.964 | 1.00(0.88-1.14) |
| FBG | 0.07 | 13.74 | <0.001 | 1.29(1.13-1.48) |
| LVEF | 0.00 | 6.10 | 0.014 | 1.00(1.00-1.00) |
| DD+ | 0.67 | 0.90 | 0.343 | 1.88(0.51-6.95) |
| TC | 0.28 | 0.23 | 0.635 | 0.88(0.51-1.51) |
| LDLC | 0.39 | 0.40 | 0.530 | 0.78(0.36-1.69) |
| ACEIs or ARBs (yes) | 1.04 | 3.63 | 0.057 | 0.14(0.02-1.06) |
| *β*-blockers (yes) | 0.58 | 0.02 | 0.880 | 1.09(0.35-3.39) |
| Statins (yes) | 1.05 | 0.80 | 0.371 | 2.55(0.33-19.88) |
| Antiplatelet drugs (yes) | 0.67 | 11.33 | <0.001 | 9.47(2.56-35.08) |

BMI: body mass index; CVDs: cardiovascular diseases; CHF: chronic heart failure; DM: diabetes mellitus; FBG: fasting blood glucose; LVEF: left ventricular ejection fraction; DD: diaphragm dysfunction; TC: total cholesterol; LDLC: low density lipoprotein cholesterol; ACEIs: angiotensin converting enzyme inhibitors; ARBs: angiotensin receptor blockers.


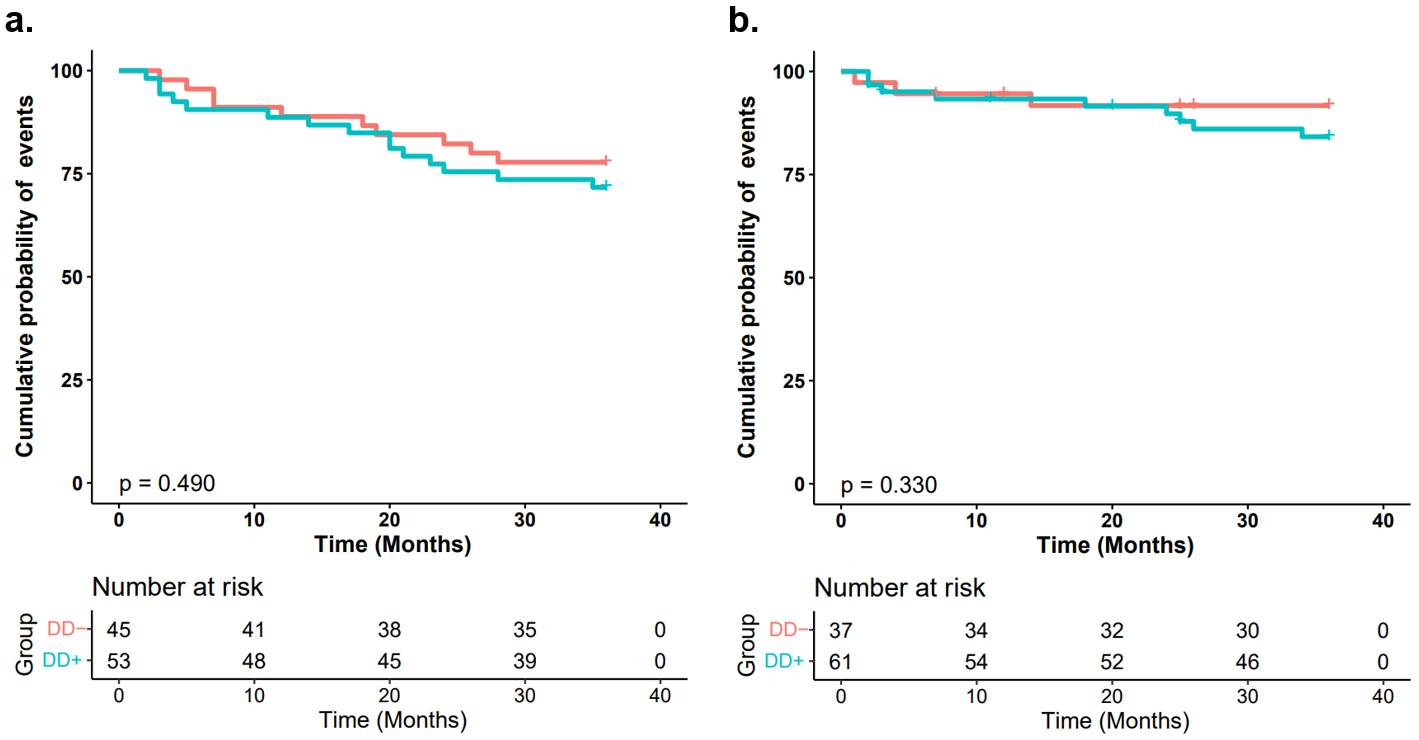


**Figure S1**

**FigureS1** Kaplan–Meier analysis by log-rank test. a. the endpoint was MACEs; b. the endpoint was MACEs+. DD+: diaphragm dysfunction(blue line); DD-: normal diaphragm function(red line).
